# Unraveling the secrets of plant roots: Simplified method for large scale root exudate sampling and analysis in *Arabidopsis thaliana*

**DOI:** 10.12688/openreseurope.15377.1

**Published:** 2023-01-18

**Authors:** Harihar Jaishree Subrahmaniam, Camilla Lind Salomonsen, Simona Radutoiu, Bodil K. Ehlers, Marianne Glasius

**Affiliations:** 1 Department of Ecoscience, Aarhus University, Aarhus, 8000 C, Denmark; 2 Department of Chemistry, Aarhus University, Aarhus, 8000 C, Denmark; 3 Department of Molecular Biology and Genetics - Plant Molecular Biology, Aarhus University, Aarhus, 8000 C, Denmark

**Keywords:** root exudates, natural variation, Arabidopsis thaliana, interdisciplinary, chemical analysis

## Abstract

**Background: **Plants exude a plethora of compounds to communicate with their environment. Although much is known about above-ground plant communication, we are only beginning to fathom the complexities of below ground chemical communication channels. Studying root-exuded compounds and their role in plant communication has been difficult due to a lack of standardized methodologies. Here, we develop an interdisciplinary workflow to explore the natural variation in root exudate chemical composition of the model plant *Arabidopsis thaliana*. We highlight key challenges associated with sampling strategies and develop a framework for analyzing both narrow and broad scale patterns of root exudate composition in a large set of natural *A. thaliana* accessions.

**Methods:** Our method involves cultivating individual seedlings *in vitro* inside a plastic mesh, followed by a short hydroponic sampling period in small quantities of ultrapure water. The mesh makes it easy to handle plants of different sizes and allows for large-scale characterization of individual plant root exudates in axenic conditions. This setup can also be easily extended for prolonged temporal exudate collection experiments. Furthermore, the short sampling time minimizes the duration of the experiment while still providing sufficient signal even with a small volume of sampling solution. We used ultra-high performance liquid chromatography coupled with quadrupole time-of-flight mass spectrometry (UHPLC-QTOF-MS) for untargeted metabolic profiling, followed by tentative compound identification using MZMine3 and SIRIUS 5 software, to capture a broad overview of root exudate composition in *A. thaliana* accessions.

**Results: **Based on 28 replicates of Columbia genotype (Col-0) compared with 10 random biological controls, MZMine3 identified 354 metabolites to be present only in Col-0 by negative ionization. Of these, 313 compounds could be annotated by SIRIUS 5 software.

**Conclusions:** The methodology developed in this study can be used to broadly investigate the role of root exudates as chemical signals in plant belowground interactions.

## Plain language summary

Plants exude many compounds to communicate with their surroundings. For decades, our understanding of this chemical communication was limited to studying the aboveground parts of plants, as roots are hidden within the soil which makes them difficult to study. We are only beginning to comprehend the complexities and importance of below ground communication channels (including plant-microbes, plant-insects, and plant-plants). Identifying the chemical compounds plant exude belowground (called root exudates) is important for us to fully comprehend their potential roles in a plant´s life. Here we developed a simplified and easy-to-manage setup for collecting and analyzing root exudates from individual *Arabidopsis thaliana* plants.

## Introduction

Plants have fascinating abilities to detect and respond to numerous chemical signals, both above and below ground, to interact with their environments ^1, 2^. While much is known about these above ground communication channels, we are only beginning to understand the complexities of below-ground plant-environment interactions, which are mediated by root exudates ^3^.

Plant-secreted secondary metabolites are a particularly intriguing fraction of root exudates, which have several hypothesized and demonstrated important functions in belowground signaling mechanisms ^1, 4, 5^. Belowground signaling has been shown to trigger a series of response mechanisms in roots including neighbor detection, recognition and response strategies for chemical defense and/or root behavioural shifts ^4, 6– 10^. Importantly, these interactions can also directly affect the above ground plant performance, directly altering plant fitness ^6, 11^.

Surprisingly, despite their demonstrated importance in mediating plant-environment interactions, we still have very little information on the identity, patterns, and levels of variation of the chemical compounds involved in belowground signaling ^2, 3^. This is partly due to the experimental as well as analytical challenges associated with trapping and identifying root-exuded chemicals, which are essential for conducting experiments of this nature. Particularly, there is no consensus on the best possible conditions and methods for isolating root exudates, exacerbated by the difficulties of standardizing methodologies for their analysis ^12^. These challenges are highlighted by multiple recent studies ^1, 12– 17^.

Besides, to make any inferences on belowground chemical communication patterns, particularly in the context of natural neighbor interactions, it is important to first gauge the individual composition and variation patterns of root exuded compounds in natural plant populations, but this has not yet been fully implemented in plant-environment interaction studies ^3^. The majority of studies aimed at root exudates often follow a targeted approach where a compound of interest is tracked for its role in driving plant behaviour using a small set of sample accessions. To date, only a few studies have aimed at identifying any broad scale patterns in root exudates (reviewed in 1, 3).

Depending on the research question, the strategy of trapping exudates and analysis has varied ^12, 13, 18^. However, to avoid major alterations to the exudate profile, which may occur due to the sorption of compounds in soil matrix or decomposition by microbes, hydroponic sterile systems are usually preferred ^12, 13^. Hydroponic collections of exudates not only minimize root damage thereby encapsulating almost all rhizodeposits, but also require fewer manipulations in downstream analysis ^12, 18^.

Here, we developed a multidisciplinary method for large scale sampling of root exudates in a hydroponic system. The method was applied for collecting root exudates of *Arabidopsis thaliana* natural accessions, where the Columbia (Col-0) accession ( CS1092) was placed as a phytometer. The method was optimized for this collection, and we demonstrate its validity by discussing the results obtained from these phytometers. The remaining data will be processed for further investigations. We made several adjustments to previous methods in order to optimize a strategy for broad scale pattern detection of root exudate chemical composition in natural accessions of *A. thaliana*. The modifications are mainly with respect to sampling method, collection period as well as chemical analysis, and are discussed in the following section. 

**(1) Sampling method:** Previous well-described hydroponic methods for root exudate sampling include setups where germinated seedlings are cultivated in liquid Murashige and Skoog´s (MS) media for weeks with continuous shaking for aeration and regular changing of nutrient solution to prevent microbial contamination ^19, 20^. Following this, hydroponic sampling is carried out ^19, 21, 22^; or mixed strategies where soil-grown plants are transferred briefly to a sample solution ^23^. Other elegant *in vitro* hydroponic setups have also been developed including Magenta boxes ^24, 25^ or 96 well PCR plates ^22^, which can be easily transferred for sampling and collecting large volumes of root exudates. However, in most cases, the exudates from multiple plants are pooled and analysed as one sample. This is due to a notion that individual roots often do not yield enough exudates, thus requiring additional steps of pooling and/or pre-concentrating the samples before analysis. However, to gauge the variation of root exudate chemical profiles of a large number of genotypes, especially for the purpose of plant communication, it is imperative to study individual exudation patterns in order to account for pattern differences due to intraspecific interactions.

Furthermore, sampling solutions in hydroponic setups often use pure distilled/demineralized water without nutrients for its compatibility with all up-concentration techniques and further analytical approaches. However, such sampling may also damage root cell membranes irreversibly due to differences in pH and/or salt concentration between growth and sampling solutions. To reduce these shock effects and capture any altered exudate patterns, it has been recommended to submerge the roots in a separate water solution briefly prior to the transfer into final sampling solution ^12^.

Finally, older plants might grow large enough to penetrate the agar layer in petriplates and form thick roots, making it difficult to remove them from the media. On the other hand, when dealing with biological/natural variation, plants of all shapes and sizes that have germinated and have developed roots large enough to exude chemical compounds, must be accounted for. Therefore, it was necessary to find a way that allows easy manipulation and handling of plants and help act as a floater for individuals when being sampled in hydroponic system. 

In this method, we grew individual *Arabidopsis thaliana* genotypes in an *in vitro* system under controlled sterile conditions, where each plant was grown inside a plastic float with a small pore size (4mm x 4mm); a material which can be easily found in laboratories, gardens or homes for garden fences and plant protection. This material can also be easily autoclaved. The mesh permits easy removal of the plant from media with minimal damage. Prior to sampling, the plant roots were submerged in MilliQ water for two minutes to absorb any erroneous exudation pattern. After this step, sampling was carried out in micro quantities of ultrapure MilliQ water. This sampling method eliminated the need for concentrating samples for further analysis.

**(2) Sampling period:** The root exudate chemical profile of plants has been demonstrated to vary with many physiological factors including the type of nutrient media, plant age, experimental setups (including abiotic and biotic components of the experiment), and even the time of day when sampling is carried out ^12, 13^. Moreover, it has also been shown that this profile can be altered with the sampling duration. Long periods of stagnant solution sampling may lead to nutrient re-uptake of some compounds, and lack of proper aeration may stress the plants leading to altered profiles ^12, 13^. In the case of *Arabidopsis thaliana,* sampling of the exudates has been carried out in between 1-6 weeks post germination ^19, 26, 27^ when the plants have reached peak vegetative state but have not yet switched to the reproductive state. Some temporal exudate collection experiments also include keeping the plants in orbital shaker with continual sample collection periods ranging between 1–7 days ^19, 22, 24, 26, 28^. It is noteworthy that in most cases, root exudates from multiple genotypes were pooled before further analysis.

However, for large scale sampling of multitudes of natural accessions, optimizing the time of collection can be tricky. This is due to their varying germination times, which may directly or indirectly impact their size. Moreover, different natural accessions may also differ in the time required to complete their life cycles, thus affecting their chemical profiles ^3^. Hence, it might be difficult to capture their natural variation of root chemical profiles throughout their life. Therefore, a setup must be imagined where continual sampling of individuals is possible. Furthermore, to control for the alteration of exudate pattern due to hypoxia, a brief time period of sampling has been recommended ^12^.

We sampled plants six weeks after germination and collected the root exudates for two hours in MilliQ water. Ultrapure water sampling solutions are endorsed for capturing polar and semi polar fractions of metabolites with minimal to no root damage ^18^ and minimizes the manipulation steps required for exudate analysis procedures ^18^. Additionally, reducing the period of sampling proved highly advantageous while dealing with hundreds of natural genotypes and was validated by still producing sufficient signal in downstream analysis.

Lastly, as found in previous experiments, we could also apply this setup for prolonged temporal root exudate collection experiments (unpublished results). Upon removal from the agar media, plants in the mesh could be easily transferred to hydroponic system in liquid media with recommended orbital shaking for long term sampling experiments (similar to 19, 22, 24, 26, 29).

**(3) Sample analysis:** The variety of root exudates and their varying chemical properties makes it challenging to analyse them. Plants produce a plethora of chemically diverse secondary metabolites, many of which exert bioactive effects on their biotic as well as abiotic environment ^22, 30, 31^. For analysing the secondary fraction of root metabolome, Liquid chromatography, in particular high-performance liquid chromatography coupled with mass spectrometry (HPLC-MS) has been broadly applied ^22, 32, 33^.

However, to date, most studies on root exudates has focused on a targeted approach wherein a known compound of interest is investigated across samples using analytical methods like spectrophotometry, ion chromatography (IC), HPLC, gas or liquid chromatography coupled with tandem mass spectrometry (GC–MS/MS, LC-MS/MS) etc. ^12^. In targeted analysis specific compound(s) or classes of compound(s) are detected and quantified using authentic standards. This type of analysis sensitively measures even minute quantities of interesting compounds and has been highly useful for investigating plant specific exudate patterns across e.g. different environments ^23^ and ages ^34^. However, taking a targeted approach limits the ability to capture any broader patterns in exudate chemical profiles or identify any novel compounds which might serve important signaling and/or response functions for a plant ^1^.

Non-targeted approaches are becoming increasingly popular in root exudate chemical analysis ^19, 22, 27^ because they allow comparisons of metabolic patterns at different conditions across samples and can be used to draw inferential hypotheses about the putative biological functions of the compound (s) tentatively characterized. Since a majority of compounds involved in plant communication remains to be discovered ^1^, an untargeted approach for characterizing the large-scale variation of exudate chemistry in natural accessions represents a first step. This data can be informative for drawing conclusions about broad scale variation patterns, which can then be used to derive hypotheses followed by multidisciplinary investigations. Furthermore, with this information, a targeted approach can also be implemented, where interesting compound(s) can be identified using authentic standards and quantified in biological samples ^35^. 

## Methods

### Plant root exudate collection

The Col-0 accessions ( CS1092) were obtained from the lab of Associate Prof. Simona Radutoiu (Dept. of Plant Molecular Biology, Aarhus University).

***Seed sterilization.*** The vacuum sterilization procedure described below was optimized for dealing with a large number of different genotypes. Accessions were surface sterilized using 30 mL of bleach (Sodium hypochlorite) and 3 mL of 37% HCl. The reaction between bleach and hydrochloric acid forms chlorine gas, which is used to sterilize the seeds in vacuum. 

The steps followed in this protocol described briefly: (1) Open Eppendorf tubes containing seeds were placed in the rack and this setup was transferred to the desiccator. (2) After connecting the vacuum tube, the hypochlorite reaction was started, and the desiccator was closed. A glove can be kept along this setup inside the desiccator to indicate vacuum levels inside the setup. (3) After the vacuum is started and the glove indicator expands, the desiccator valve is closed to shut down the vacuum while the chlorine gas generated in the reaction can sterilize the seeds. (4) After 10–15 minutes, desiccator valve is slowly opened to relieve air pressure and seed vials can be removed. The seeds were taken to back to the sterile benches and kept with open lids for 10 minutes to remove any extra sterilization gas.

***Plant growth setup.*** For *in vitro* cultivation of *Arabidopsis thaliana* accessions, we used half-strength Murashige and Skoog´s medium with Gamborg´s Vitamins (Sigma Aldrich) with 0.8% plant agar (Sigma Aldrich). This nutrient solution was prepared and poured into 90mm petri dishes containing two compartments (Fisher Scientific).

**Figure 1.**
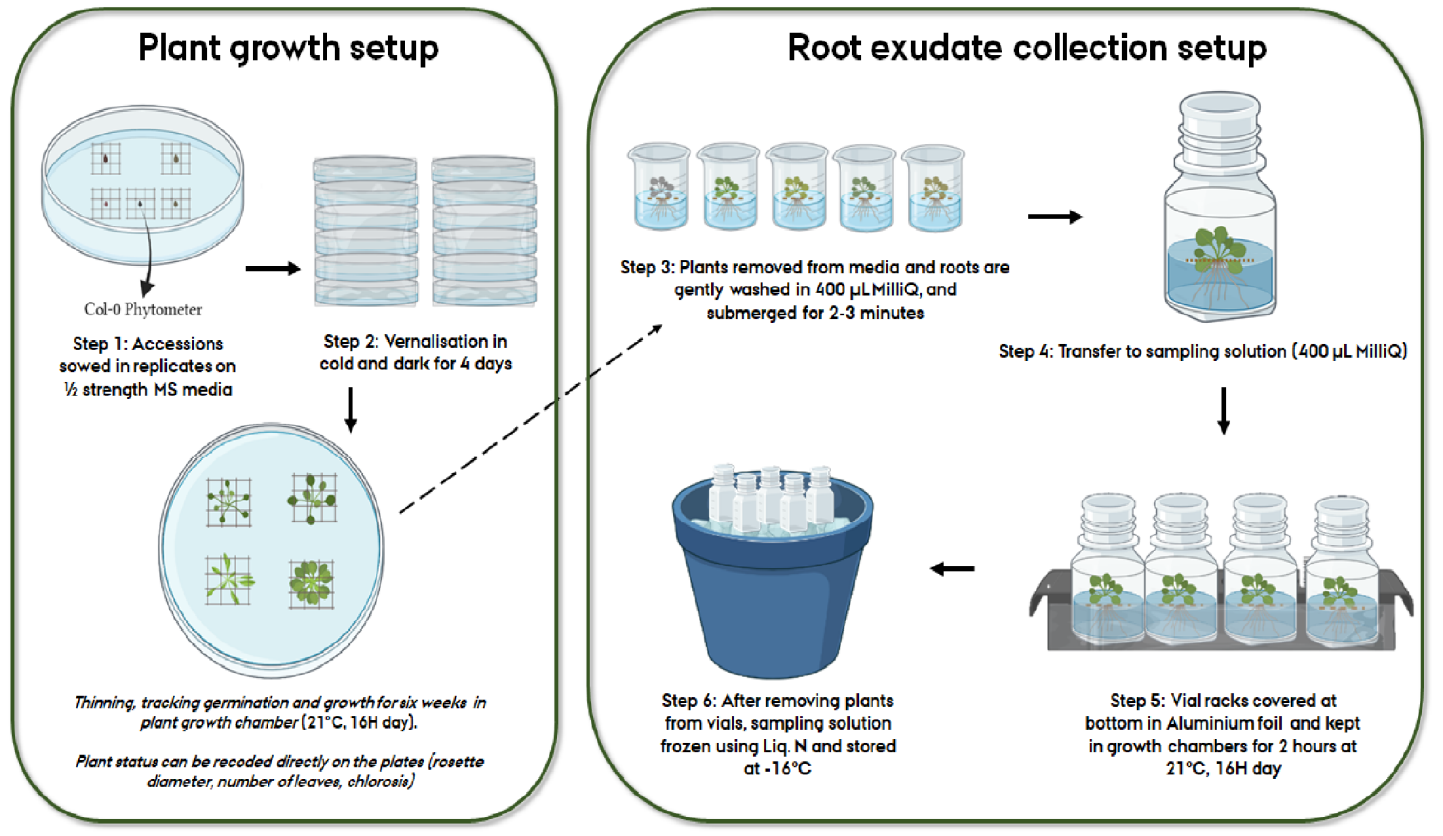
Workflow representing the various steps followed for root exudate collection of *Arabidopsis thaliana* accessions.

For ease of handling individual plants of varying sizes arising due to because of natural/experimental variation, and for creating a “floater” which allows easy root access when being sampled in liquid media, an autoclaved plastic mesh was placed in each unique position within the petri plate. Using a sterile plastic toothpick, two sterilized seeds were placed at the center of each mesh, with one plate having 4-5 unique individual replicates/genotypes (Step 1, Figure 1). After marking the positions of genotypes on the plates, the plates were secured with micropore tape. All these steps were performed under sterile conditions. For vernalization (Step 2, Figure 1), petri plates were covered in Aluminum foil and kept in a cold room (at 4°C) for four days after which they were transferred to growth chambers at 21°C with 16H day. The plates were checked daily, and individual germination dates were noted. Within 2–3 days post germination, the plants were thinned so only one plant remained within each mesh. The location of the plates was shifted every 2–3 days to minimize position effects. Phenotypic measures including rosette diameter, number of leaves and their extent of chlorosis ( Figure 1), could be scored directly on the plates.

***Sample collection.*** Six-week-old plants were gently removed from agar media using sterilized tweezers (Step 3, Figure 1). Handling the mesh instead of plant minimizes the damage caused to the plant during the transfer. The agar sticking to the root was gently removed with a sterilized paint brush. Following this, the plants were transferred to individual beakers with a cleaning solution of 400µL MilliQ water for about two minutes (Step 3, Figure 1). This step is recommended for capturing altered exudates due to the initial shock of transferring to liquid media and is also useful in further removal of agar residues from the roots. The plants were then transferred to 400µL MilliQ sampling solution in 15mL glass vials and placed in racks (Step 4, Figure 1). For the sake of sterility, the vial lids were closed, and aeration was prevented. All these steps were performed inside a flow bench (LaboGene ScanLaf Model Mars). The racks were removed from the flow bench, and bottom half of the racks was covered in Aluminum foil to mimic the dark conditions roots experience in nature. The setup was then transferred to a growth chamber (21°C) for two hours (Step 5, Figure 1). After this, the setup was brought back to the flow benches where the plants were gently removed from the sampling solution. The vials with sampling solution were then frozen in liquid nitrogen and subsequently stored at -18°C (Step 6, Figure 1).

### Root exudate analysis

The method for root exudate analysis has been optimized for both targeted and untargeted analysis of root exudates and has a run time for each sample to 28 minutes. The details of this method will be discussed in a forthcoming publication from the group but is described briefly below. 

***Preparation of samples for UHPLC analysis.*** Prior to sample preparation, sample solutions in vials were allowed to thaw at room temperature. An internal standard of ketopinic acid (10µg/mL in MilliQ) was prepared for spiking the samples. This allows checking for injection efficiency and gauging sample signal strength in LC-MS analysis. 180µL exudate sample was added to 20µL of internal standard and filtered using 0.22μm Polytetrafluoroethylene (PTFE) hydrophobic syringe filters and finally transferred to 2mL vials with inserts. 

***HPLC-MS analysis.*** The samples were analysed on a Thermo UltiMate 3000 UHPLC coupled to a quadrupole time-of-flight mass spectrometer, qTOF (Bruker, Compact). The column was a Waters Acquity UHPLC HSS T3 column, which has a length of 100 mm and a diameter of 2.1 mm and with a particle diameter of 1.8μm. The column temperature was 45°C. The mobile phase consisted of 0.1% acetic acid (AA), in MilliQ-water (eluent A) and 0.1% AA in acetonitrile (eluent B). The flow rate was 0.200 mL min ^−1^ for the entire analysis, and the injection volume was 2μL. The UHPLC was coupled to the qTOF, through an electrospray ionization (ESI) inlet in negative ionization mode. Prior to each run, the MS was calibrated using 5 mM sodium acetate solution (HCOONa). Operating settings for the MS were as follows: the capillary voltage was 4.5 kV, the drying gas (N _2_) was 5.5L min ^−1^, the nebulizer pressure was 2.5 bar, the capillary temperature was 200°C, and the scanning interval was 50-1000 m/z. Data was acquired and processed using Bruker software (DataAnalysis) followed by MZMine3 and SIRIUS 5.

Data obtained was first analysed on the proprietary software Bruker Compass DataAnalysis 4.3, where background noise levels, signal intensities, signal-to-noise ratios etc. can be deeply investigated. The open access softwares OpenChrom and MZMine are some freely available alternatives for such manual examination of the data. MZMine 3 is an open-source software for mass-spectrometry data processing, especially from LC-MS data. Peak alignment and noise filtering were carried out with MzMine3. The noise threshold was estimated as 1.0E ^2^, and the minimum feature height threshold to be considered a true signal was kept as at least three times the noise level. Applying appropriate filters from control peaks, chromatogram peaks were then aligned across samples. All the relevant features identified were further analysed with SIRIUS 5 software another open access Java software for analysing metabolites from tandem mass spectrometry data ^36, 37^. SIRIUS provides a fragmentation tree to each relevant peak (feature), describing the fragmentation reaction from each molecule from the ESI. The CSI:FingerID feature can then be used to search in molecular structure databases from the internet for tentative identification of compounds ^38^.

## Results

**Figure 2.**
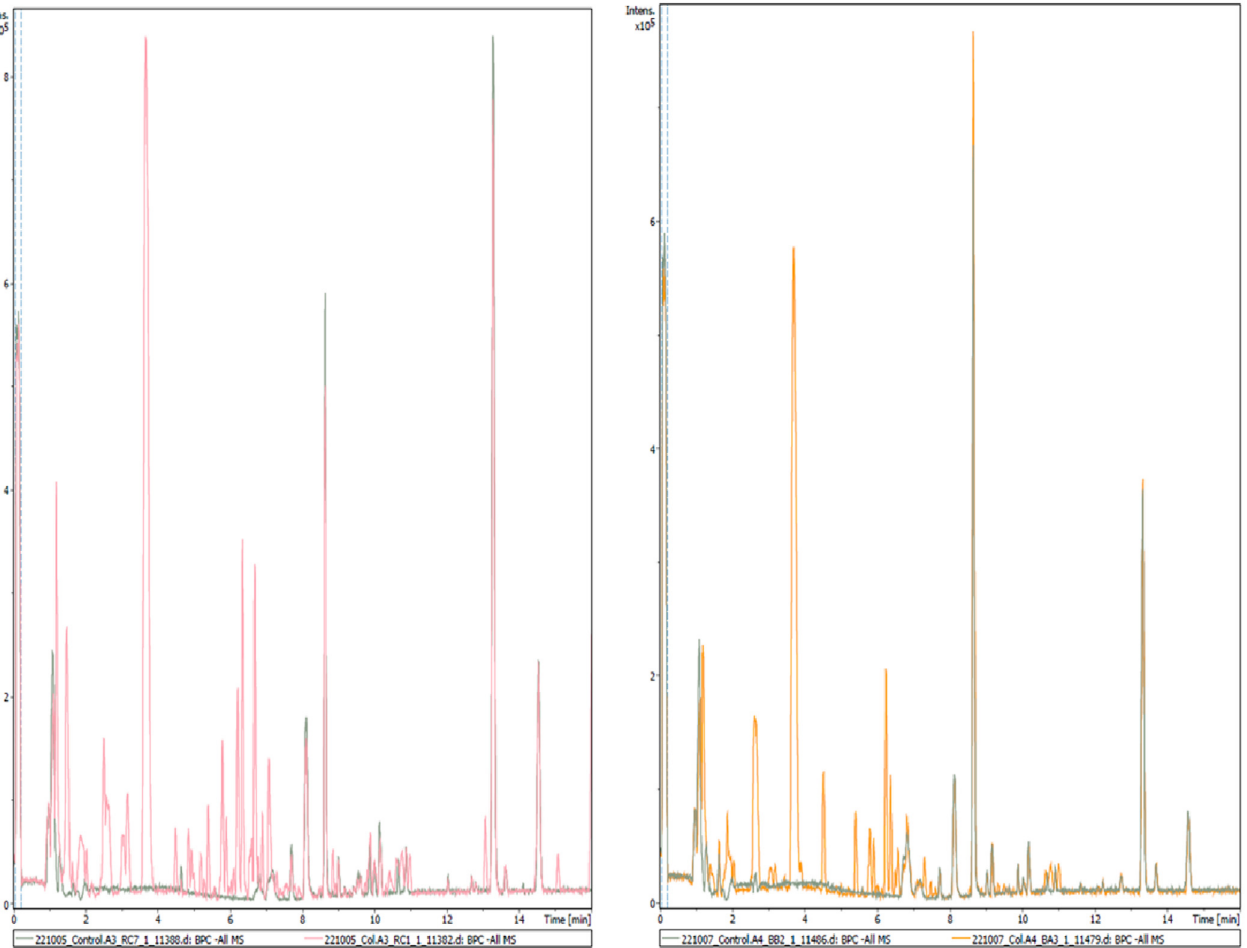
Representative chromatograms of two different Col-0 accessions (m/z 121–832, rt 0.99-15 minutes) obtained from root exudates overlaid over blank samples by UHPLC/ESI-QTOFMS in negative ion mode. A total of 354 high intensity peaks are identified to be present only in Col-0 accessions and are listed in Supplementary Table 1 (see *Extended data* ^39^).

Figure 2 shows the chromatograms obtained from untargeted analysis in negative ionization mode of Col-0 accessions using the above-described method overlaid over biological controls (in green). Using 28 replicates of Col-0 samples, MZMine3 identified and aligned 378 features/chemical compounds, whereas the 10 random biological controls consisted of 68 features (see *Underlying data* ^39^). After subtracting the features present in biological controls, we identified 354 metabolic features present only in Col-0 accessions (see Supplementary Table 1 in *Extended data* ^39^). Following this, the CSI:FingerID web service feature of SIRIUS 5 classified 313 compounds (around 88% of the features identified) present in the samples. Their chemical annotations are listed in Supplementary Table 2 (see *Extended data* ^39^).

## Discussion

Root exudates are substantially hard to study, yet the importance of root-secreted signaling chemicals in mediating plant interactions is no longer debatable. So far, the methodologies available to trap and identify root-secreted signaling chemicals has remained a serious problem ^40^. This has limited our abilities to identify any patterns of their natural variation on a large sample of accessions, and thus, their role in driving belowground communications remains to be elucidated.

 The method developed here was optimized for large scale root exudate sampling of *A. thaliana*, natural accessions. With this method, by reducing time of collection and omitting orbital shaking, we aimed to diminish the shock effects experienced by plants during transfer from solid to liquid media. Furthermore, faster chemical analysis time is another significant improvement when dealing with hundreds of samples. Even with minute sampling solutions, the method yielded sufficient signals for the LC analysis, which was sensitive enough to capture micro quantities of several metabolites. This is validated by the number of secondary metabolites identified in the analysis. Moreover, SIRIUS 5 was able to annotate a substantial number of features (listed in Supplementary Table 2 in *Extended data* ^39^), and this information can be used to draw broad patterns and make inferential hypotheses to be validated by complementary approaches. Our method is valuable for quick pattern assessment experiments of a large samples of accessions for the purpose of studying plant-environment interactions (including but not limited to plant-microbes, plant-insects, and plant-plants).

Even though hydroponic root exudate collection is not a perfect solution, as we cannot fully mimic the conditions roots may experience in nature ^12^, these methods are still highly useful setups that allow capturing exudates in sterile environments ^13^. In this method, using a cost-effective plastic floater, plants were easily managed during the entire growth and hydroponic sampling steps. Furthermore, this floating setup could be translated into longer temporal collection experiments as in similar experiments conducted on *A. thaliana* ^19, 24^. Using meshes of different pore sizes, one can optimize this setup for root exudate collection and analysis of a large number of individual genotypes from other plant species.

Verifying the biochemical functions of these metabolites is without a doubt, a mammoth feat, requiring an interdisciplinary approach. Analysing root exudates in axenic conditions gives a broad overview of its chemical composition, and they can be further validated for their roles using complementary methodologies spanning multiple disciplines. For example, compounds tentatively identified by SIRIUS 5, can be quantified using a targeted approach if authentic standards are available. Many root exudate studies have previously confirmed an altered pattern of root behaviour once their ability to sense the exudates of neighboring plants are removed ^3^. Targeting specific components of exudates for validating this behaviour could also present an interesting next step. Deep investigation into the biochemical pathways of these metabolites by creating gene knockouts could also reveal their exact roles in plant behaviour modulation.

Studying root exudate chemical signals mediating belowground interactions are important not only from an ecological context but also from an applied perspective in agriculture and forestry ^1^. Root-secreted compounds are part of a complex interaction network incorporating other inter/intraspecific neighbours, bacteria, fungi and invertebrates etc., where each interacting plant might receive individual signals from multiple sources, making this exceedingly difficult to dissect. Hence, methodologies are needed to first simplify studying these interactions and identifying ecologically relevant biochemical communication channels ^41^, which can be ultimately applied in agricultural context ^1^. Scrutinizing the variation in chemical profiles of interacting partners remains as essential first step which has been largely overlooked in root exudate studies ^13^, further hindered by following a targeted approach to identify already known compounds.

The method developed here takes a step forward in overcoming limitations from previously developed hydroponic systems. By developing an easy-to-manage setup for large scale sampling, we detected many interesting secondary metabolites in Col-0, which will be used for further investigations using population genomics, evolutionary ecology and analytical chemistry approaches to answer questions relating to their roles in plant-environment interactions.

## Ethics and consent

Ethical approval and consent were not required.

## Data Availability

Zenodo: Unraveling the secrets of plant roots: Simplified method for large scale root exudate sampling and analysis in Arabidopsis thaliana. https://doi.org/10.5281/zenodo.7338812 ^39^. This project contains the following underlying data: Raw datafiles 10112022.xlsx: The raw datasets obtained from MZMine 3 analysis, which contains aligned features of Columbia genotypes (Sheet1) as well as control samples (Sheet2). The dataset consists of *feature (row)ID, average (mass-to-charge ratios) m/z* and *retention times (RT)* across samples for individual features. It also includes sample-specific information including *feature status, name, m/z, RT, feature peak height,* and *area*. We also include a filtered datasheet excluding the features obtained in control as well as samples (Sheet3). Zenodo: Unraveling the secrets of plant roots: Simplified method for large scale root exudate sampling and analysis in Arabidopsis thaliana. https://doi.org/10.5281/zenodo.7338812 ^39^. This project contains the following extended data: Supplementary tables-14112022.xlsx: The 354 metabolites obtained after filtering out control features with their *mass-to-charge ratios* and *retention times* (Supplementary Table 1). The 313 features identified by SIRIUS are listed with their *mass-to-charge ratios, retention times, chemical formula, chemical annotations,* and *probabilities scored by SIRIUS* in Supplementary Table 2. Data are available under the terms of the Creative Commons Attribution 4.0 International license (CC-BY 4.0).
