# Unraveling the secrets of plant roots: Simplified method for large scale root exudate sampling and analysis in *Arabidopsis thaliana*

**DOI:** 10.12688/openreseurope.15377.2

**Published:** 2023-07-05

**Authors:** Harihar Jaishree Subrahmaniam, Camilla Lind Salomonsen, Simona Radutoiu, Bodil K. Ehlers, Marianne Glasius

**Affiliations:** 1 Department of Ecoscience, Aarhus University, 8000 Aarhus C, Denmark; 2 Department of Chemistry, Aarhus University, 8000 Aarhus C, Denmark; 3 Department of Molecular Biology and Genetics - Plant Molecular Biology, Aarhus University, 8000 Aarhus C, Denmark

**Keywords:** root exudates, natural variation, Arabidopsis thaliana, interdisciplinary, chemical analysis

## Abstract

**Background: **Plants exude a plethora of compounds to communicate with their environment. Although much is known about above-ground plant communication, we are only beginning to fathom the complexities of below-ground chemical communication channels. Studying root-exuded compounds and their role in plant communication has been difficult due to the lack of standardized methodologies. Here, we develop an interdisciplinary workflow to explore the natural variation in root exudate chemical composition of the model plant *Arabidopsis thaliana*. We highlight key challenges associated with sampling strategies and develop a framework for analyzing both narrow- and broad-scale patterns of root exudate composition in a large set of natural *A. thaliana* accessions.

**Methods:** Our method involves cultivating individual seedlings *in vitro* inside a plastic mesh, followed by a short hydroponic sampling period in small quantities of ultrapure water. The mesh makes it easy to handle plants of different sizes and allows for large-scale characterization of individual plant root exudates under axenic conditions. This setup can also be easily extended for prolonged temporal exudate collection experiments. Furthermore, the short sampling time minimizes the duration of the experiment while still providing sufficient signal even with small volume of the sampling solution. We used ultra-high performance liquid chromatography coupled with quadrupole time-of-flight mass spectrometry (UHPLC-QTOF-MS) for untargeted metabolic profiling, followed by tentative compound identification using MZmine3 and SIRIUS 5 software, to capture a broad overview of root exudate composition in *A. thaliana* accessions.

**Results: **Based on 28 replicates of the Columbia genotype (Col-0) compared with 10 random controls, MZmine3 identified 354 metabolites to be present only in Col-0 by negative ionization. Of these, 254 compounds could be annotated by SIRIUS 5 software.

**Conclusions:** The methodology developed in this study can be used to broadly investigate the role of root exudates as chemical signals in plant belowground interactions.

## Plain language summary

Plants exude many compounds to communicate with their surroundings. For decades, our understanding of this chemical communication was limited to studying the aboveground parts of plants, as roots are hidden within the soil, which makes them difficult to study. We are only beginning to comprehend the complexities and importance of below ground communication channels (including plant-microbes, plant-insects, and plant-plants). Identifying the chemical compounds plant exude belowground (called root exudates) is important for us to fully comprehend their potential roles in a plant´s life. Here, we developed a simplified and easy-to-manage setup for collecting and analyzing root exudates from individual *Arabidopsis thaliana* plants.

## Introduction

Plants have fascinating abilities to detect and respond to numerous chemical signals, both above and below ground, to interact with their environments ^1, 2^. While much is known about these above-ground communication channels, we are only beginning to understand the complexities of below-ground plant-environment interactions, which are mediated by root exudates ^3^.

Plant-secreted secondary metabolites are a particularly intriguing fraction of root exudates, which have several hypothesized and demonstrated important functions in belowground signaling mechanisms ^1, 4, 5^. Belowground signaling has been shown to trigger a series of response mechanisms in roots, including neighbor detection, recognition and response strategies for chemical defense and/or root behavioral shifts ^4, 6– 10^. Importantly, these interactions can also directly affect the aboveground plant performance, directly altering plant fitness ^6, 11^.

Surprisingly, despite their demonstrated importance in mediating plant-environment interactions, we still have very little information on the identity, patterns, and levels of variation of the chemical compounds involved in belowground signaling ^2, 3^. This is partly due to the experimental and analytical challenges associated with trapping and identifying root-exuded chemicals, which are essential for conducting experiments of this nature. In particular, there is no consensus on the best possible conditions and methods for isolating root exudates, exacerbated by the difficulties of standardizing methodologies for their analysis ^12^. These challenges are highlighted by multiple recent studies ^1, 12– 17^.

Besides, to make any inferences on belowground chemical communication patterns, particularly in the context of natural neighbor interactions, it is important to first gauge the individual composition and variation patterns of root exuded compounds among natural plant populations. This extensive sampling approach, which has not yet been fully implemented in plant-environment interaction studies ^3^, requires a comprehensive methodology that ensures both thoroughness and time efficiency. Additionally, most studies aimed at root exudates often follow a targeted approach where a compound of interest is tracked for its role in driving plant behaviour using a small set of sample accessions. To date, only a few studies have aimed at identifying any broad-scale patterns in root exudates (reviewed in 1, 3).

Depending on the research question, the strategy of trapping exudates and analysis has varied ^12, 13, 18^. However, to avoid major alterations to the exudate profile, which may occur due to the sorption of compounds in soil matrix or decomposition by microbes, hydroponic sterile systems are usually preferred ^12, 13^. Hydroponic collections of exudates not only minimize root damage, thereby encapsulating almost all rhizodeposits, but also require fewer manipulations in downstream analysis ^12, 18^.

Here, we developed a multidisciplinary method for large-scale sampling of root exudates in a hydroponic system. The method was applied for collecting root exudates from natural accessions of *Arabidopsis thaliana*, where the Columbia (Col-0) accession ( CS1092) was placed as a phytometer. The method was optimized for this collection, and we demonstrate its validity by discussing the results obtained from these phytometers. The remaining data will be processed for further investigations. We made several adjustments to previous methods to refine a strategy for broad-scale pattern detection of root exudate chemical composition in natural accessions of *A. thaliana*. The modifications are mainly with respect to sampling method, collection period as well as chemical analysis, and are discussed in the following section.

**(1) Sampling method:** Previous studies have some well-described hydroponic methods for root exudate sampling including setups where germinated seedlings are cultivated in liquid Murashige and Skoog´s (MS) media for weeks with continuous shaking for aeration and regular changing of nutrient solution to prevent microbial contamination ^19, 20^. Following this, hydroponic sampling was carried out as in 19, 21, 22. Some studies employed mixed strategies where soil-grown plants were transferred briefly to a sample solution ^23^. Other elegant *in vitro* hydroponic setups have also been developed where sampling was carried out using Magenta boxes ^24, 25^ or 96 well PCR plates ^22^, which can be easily transferred for sampling and collecting large volumes of root exudates. However, in most cases, the exudates from multiple plants were pooled and analyzed as one sample. This is due to the notion that individual roots often do not yield enough exudates, thus requiring additional steps of pooling samples from many plants and/or pre-concentrating the samples before analysis.

Furthermore, sampling solutions in hydroponic setups often use pure distilled/demineralized water without nutrients for its compatibility with all up-concentration techniques and further analytical approaches. However, it is important to note that such sampling may cause stress to plants and damage root cell membranes irreversibly due to differences in pH and/or salt concentration between growth and sampling solutions. To reduce these shock effects and capture any altered exudate patterns, it has been recommended to briefly submerge the roots in a separate water solution before transfer into the final sampling solution ^12^.

Finally, older plants might grow large enough to penetrate the agar layer in petri plates and form thick roots, making it difficult to remove them from the media. On the other hand, when dealing with biological/natural variation, plants of all shapes and sizes that have germinated and have developed roots large enough to exude chemical compounds, must be accounted for. Therefore, it was necessary to find a way that allows easy manipulation and handling of plants and helps act as a floater for individuals when being sampled in a hydroponic system.

This method was fine-tuned for large scaling sampling of natural genotypes, where, we grew individual *Arabidopsis thaliana* genotypes in an *in vitro* system under controlled sterile conditions. Each plant was grown inside a plastic float with a small pore size (4mm x 4mm; made of polypropylene); a material that can be easily found in laboratories, gardens or homes for garden fences and plant protection. This material can also be easily autoclaved. The mesh permits easy removal of the plant from media with minimal damage. Before sampling, the plant roots were submerged in MilliQ water for two minutes to absorb any extraneous exudation pattern caused by stress. After this step, sampling was carried out in microquantities of ultrapure MilliQ water. This method thus allowed sampling of a large number of individual plants and eliminated the need for concentrating samples for further analysis.

**(2) Sampling period:** The root exudate chemical profile of plants has been demonstrated to vary with many physiological factors including the type of nutrient media, plant age, experimental setups (including abiotic and biotic components of the experiment), and even the time of day when sampling is carried out ^12, 13^. Moreover, it has also been shown that this profile can be altered with the sampling duration. Long periods of stagnant solution sampling may lead to nutrient re-uptake of some compounds, and lack of proper aeration for extended times may stress the plants leading to altered profiles ^12, 13^. In the case of *Arabidopsis thaliana,* previous studies have included sampling of the exudates between 1 and 6 weeks post germination ^19, 26, 27^ when the plants have reached peak vegetative state but have not yet switched to reproductive state. Some temporal exudate collection experiments also included keeping the plants in an orbital shaker with continual sample collection periods ranging between 1 and 7 days ^19, 22, 24, 26, 28^.

However, for large-scale sampling of multitudes of natural accessions, streamlining the time of collection can be tricky. This is due to their varying germination times, which may directly or indirectly impact their size. Moreover, different natural accessions may also differ in the time required to complete their life cycles, thus affecting their chemical profiles ^3^. Hence, it might be difficult to capture their natural variation of root chemical profiles throughout their life. Therefore, a setup must be imagined where continual sampling of individuals is possible.

In this method, to capture a snapshot of the chemical composition of root exudates, we sampled plants six weeks after germination and collected the root exudates for two hours in MilliQ water. Despite the potential additional stress imposed during such sampling, ultrapure water sampling solutions are endorsed for capturing polar and semi polar fractions of metabolites with minimal to no root damage ^18^ and minimizes the manipulation steps required for exudate analysis procedures ^18^.

The two-hour sampling duration was chosen to minimize the sampling time and potential underestimation of total organic carbon ^29^. Additionally, this duration is less likely to be affected by metabolite re-uptake ^30^. Our preliminary experiments with time exposure (unpublished results) also supported the suitability of a two-hour collection time for root exudates in *Lotus japonicus* genotypes. Moreover, the exudate collection under sterile/anaerobic conditions for this short duration has been validated in recent studies involving different plant species ^29– 32^. Reducing the sampling period proved highly advantageous when handling hundreds of natural genotypes while still producing a sufficient signal in downstream analysis.

Additionally, as found in previous experiments, we could also apply this setup for prolonged temporal root exudate collection experiments (unpublished results). Upon removal from the agar media, plants in the mesh could be easily transferred to a hydroponic system in suitable amount of liquid media with recommended orbital shaking for long-term sampling experiments (similar to 19, 22, 24, 26, 33).

**(3) Sample analysis:** The variety of root exudates and their varying chemical properties make it challenging to analyze them. For analysing the secondary fraction of root metabolome, liquid chromatography, in particular high-performance liquid chromatography coupled with mass spectrometry (HPLC-MS) has been broadly applied ^22, 34, 35^.

However, to date, most studies on root exudates have focused on a targeted approach in which a known compound of interest is investigated across samples using analytical methods such as spectrophotometry, ion chromatography (IC), HPLC, gas or liquid chromatography coupled with tandem mass spectrometry (GC–MS/MS, LC-MS/MS) etc. ^12^. In targeted analysis, specific compound(s) or classes of compound(s) are detected and quantified using authentic standards. This type of analysis sensitively measures even minute quantities of interesting compounds and has been highly useful for investigating plant specific exudate patterns across different environments ^23^ and ages ^36^. However, taking a targeted approach limits the ability to capture any broader patterns in exudate chemical profiles or identify any novel compounds that might serve important signaling and/or response functions for a plant ^1^.

Non-targeted approaches are becoming increasingly popular in root exudate chemical analysis ^19, 22, 27^ because they allow comparisons of metabolic patterns at different conditions across samples and can be used to draw inferential hypotheses about putative biological functions of the compound (s) tentatively characterized. Since the majority of compounds involved in plant communication remain to be discovered ^1^, an untargeted approach for characterizing the large-scale variation of exudate chemistry in natural accessions represents a first step. These data can be informative for drawing conclusions about broad-scale variation patterns, which can then be used to derive hypotheses followed by multidisciplinary investigations. Furthermore, with this information, a targeted approach can also be implemented, where interesting compound(s) can be identified using authentic standards and quantified in biological samples ^37^.

To enable the identification, quantification, and comprehensive analysis of metabolites in untargeted analysis of a large dataset encompassing multiple plants, we used a synergistic approach employing two robust open-access software tools: MZmine 3 and SIRIUS 5. This combination of tools provides powerful capabilities for processing and analyzing the LCMS data, ensuring an in-depth exploration of the metabolomic landscape ^38^.

## Methods

### Plant root exudate collection

The Col-0 accessions ( CS1092) were obtained from the lab of Associate Prof. Simona Radutoiu (Dept. of Plant Molecular Biology, Aarhus University). The methodology was designed to refine the process of root exudate collection from over 100 natural accessions, with Col-0 used as the phytometer. To ensure experimental control, plants were divided into seven batches with one Col-0 and one control (a mesh without plant) per batch. Controls were treated identically to plants throughout the experiment, thus serving as baseline references. The experiment was conducted in five replicates, with each replicate consisting of seven batches, mounting to 35 Col-0 phytometers. With synchronized procedures within each batch, we reduced the effects of confounding factors. Sample collection occurred during a specified period to minimize diurnal variations.

***Seed sterilization.*** The vacuum sterilization procedure described below was adjusted for dealing with many different genotypes. Accessions were surface sterilized using 30 mL of sodium hypochlorite (bleach) and 3 mL of 37% HCl. The reaction between bleach and hydrochloric acid form chlorine gas, which is used to sterilize the seeds in vacuum.

The steps followed in this protocol are described briefly: (1) Open Eppendorf tubes containing seeds were placed in the rack and this setup was transferred to the desiccator. (2) After connecting the vacuum tube, the hypochlorite reaction was started, and the desiccator was closed. A glove can be kept along this setup inside the desiccator to indicate vacuum levels inside the setup. (3) After the vacuum is started, the air inside the desiccator is removed reducing the pressure, thus making the glove indicator expand ^39^. Once the glove expands the desiccator valve was closed to shut down the vacuum while the chlorine gas generated in the reaction sterilized the seeds. (4) After 10–15 minutes, desiccator valve was slowly opened to relieve air pressure after which seed vials could be removed. The seeds were taken back to the sterile benches and kept with open lids for 10 minutes to remove any extra sterilization gas.

***Plant growth setup.*** For *in vitro* cultivation of *Arabidopsis thaliana* accessions, we used half-strength Murashige and Skoog´s medium with Gamborg´s Vitamins (Sigma Aldrich) and 0.8% plant agar (Sigma Aldrich). This nutrient solution was prepared and poured into 90mm Petri dishes containing two compartments (Fisher Scientific).

**Figure 1.**
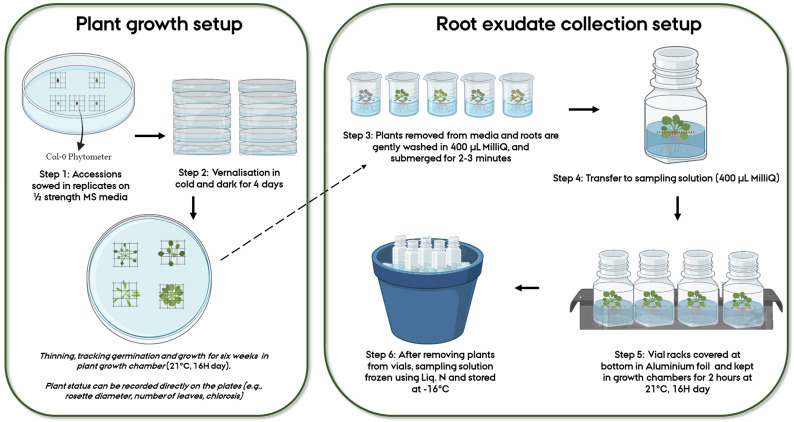
Workflow representing the various steps followed for root exudate collection of *Arabidopsis thaliana* accessions.

For ease of handling individual plants of varying sizes due to biological variation, and for creating a “floater” allowing easy root access when sampling in liquid media, an autoclaved plastic mesh was placed in each unique position within the Petri plate. Using a sterile plastic toothpick, two sterilized seeds were placed at the center of each mesh, with one plate having 4-5 unique individual replicates/genotypes (Step 1, Figure 1). After marking the positions of genotypes on the plates, the plates were secured with micropore tape. All these steps were performed under sterile conditions. For vernalization (Step 2, Figure 1), Petri plates were covered in aluminum foil and kept in a cold room (at 4°C) for four days after which they were transferred to growth chambers at 21°C with 16H day. The plates were checked daily, and individual germination dates were noted. Within 2–3 days post germination, the plants were thinned so that only one plant remained within each mesh. The location of the plates was shifted every 2–3 days to minimize position effects. Phenotypic measures including rosette diameter, the number of leaves and extent of chlorosis ( Figure 1), could be scored directly on the plates.

***Sample collection.*** Six-week-old plants were gently removed from agar media using sterilized tweezers (Step 3, Figure 1). The transfer of plants from the plates to the sampling solution took approximately 5–6 minutes per plant, depending on the plant sample size. All plants from a plate were treated at a time. Since the plants in our study were small and easy to remove from the agar and clean, this step was relatively quick and straightforward. Handling the mesh instead of the plant minimizes the damage caused to the plant during the transfer. The agar sticking to the root was gently removed with a sterilized paint brush. This step took approximately 2–3 minutes per plant (depending on the size of the plant). Following this, the plants were transferred to individual beakers with a cleaning solution of 400 µL MilliQ waterfor about two minutes (Step 3, Figure 1). This step is recommended for capturing any impertinent exudates due to the initial shock/stress of transferring to liquid media and is also useful in further removal of agar residues from the roots. The plants were then transferred to already prepared sampling vials of 400 µL MilliQ water in 20 mL cylindrical glass vials (27.5 mm diameter x 57 mm height), where the roots were submerged in the solution and the above ground part remained floating, and placed in racks (Step 4, Figure 1). For the sake of sterility, the vial lids were closed, and aeration was prevented. Control treatments were similarly sampled. All these steps were performed inside a flow bench (LaboGene ScanLaf Model Mars). The racks were removed from the flow bench, and the bottom half of the racks was covered in aluminum foil to mimic the dark conditions roots experience in nature. The setup was then transferred to a growth chamber (21°C) for two hours (Step 5, Figure 1). During the experiment, up to 60 samples could be handled in one sitting. The growth chamber facility was conveniently located in the same building as the laminar flow chambers, facilitating the transfer of plants between the two environments without prolonged exposure to external environmental conditions. After this, the setup was brought back to the flow benches where the plants were gently removed from the sampling vials (1–2 minutes per vial). The vials were then immediately frozen in liquid nitrogen and subsequently stored at -18°C (Step 6, Figure 1).

### Root exudate analysis

The method for root exudate analysis has been optimized for both targeted and untargeted analysis of root exudates and has a run time of 28 minutes for each sample in negative ionization mode. The details of this method will be discussed in a forthcoming publication from the group but is described briefly below.

***Preparation of samples for UHPLC analysis.*** Before sample preparation, sample solutions in vials were allowed to thaw at room temperature. An internal standard of ketopinic acid (10µg/mL in MilliQ water) was prepared for spiking the samples. This allows checking for injection efficiency and gauging sample signal strength in LC-MS analysis. 180 µL exudate sample was added to 20 µL of internal standard and filtered using 0.22 μm Polytetrafluoroethylene (PTFE) hydrophobic syringe filters and finally transferred to 2 mL vials with inserts.

***HPLC-MS analysis.*** The samples were analyzed on a Thermo UltiMate 3000 UHPLC coupled to a quadrupole time-of-flight mass spectrometer, qTOF (Bruker, Compact). The column was a Waters Acquity UHPLC HSS T3 column, which had a length of 100 mm and a diameter of 2.1 mm and a particle diameter of 1.8 μm. The column temperature was 45°C. The mobile phase consisted of 0.1% acetic acid (AA), in MilliQ-water (eluent A) and 0.1% AA in acetonitrile (eluent B). The flow rate was 0.200 mL min ^−1^ for the entire analysis, and the injection volume was 2μL. The UHPLC was coupled to the qTOF, through an electrospray ionization (ESI) inlet in the negative ionization mode. Prior to each run, the MS was calibrated using 5 mM sodium acetate solution (HCOONa). Operating settings for the MS were as follows: the capillary voltage was 4.5 kV, the drying gas (N _2_) was 5.5L min ^−1^, the nebulizer pressure was 2.5 bar, the capillary temperature was 200°C, and the scanning interval was 50–1000 m/z. Data were acquired and processed using Bruker software (DataAnalysis) followed by MZmine3 and SIRIUS 5.

The data obtained was first analyzed using the proprietary software Bruker Compass DataAnalysis 4.3, where background noise levels, signal intensities, signal-to-noise ratios, etc. can be investigated in depth. The open access software OpenChrom and MZmine are some freely available alternatives for such manual examination of the data. MZmine 3 is an open-source software for mass-spectrometry data processing, especially from LC-MS data. Peak alignment and noise filtering were carried out with Mzmine3. The noise threshold was estimated as 1.0E ^2^, and the minimum feature height threshold to be considered a true signal was kept at least three times the noise level. Applying appropriate filters from control peaks, the chromatogram peaks were then aligned across samples. All the relevant features identified were further analyzed with SIRIUS 5 software another open access Java software for analyzing metabolites from tandem mass spectrometry data ^40, 41^. SIRIUS provides a fragmentation tree to each relevant peak (feature), describing the fragmentation reaction from each molecule from the ESI. The CSI:FingerID feature can then be used to search in molecular structure databases from the internet for tentative identification of compounds ^42^.

Notably, SIRIUS 5's natural product classification (NPC) allows clustering of identified compounds into class, superclass, and pathway categories, which significantly enhances our understanding of the biological interpretations of the metabolites ^43^. In the analysis conducted using both positive and negative ionization modes, a higher number of features were identified in the negative mode (379) compared to the positive mode (76). As a result, we will focus on the negative mode results in this context to highlight its capability in detecting and identifying a significant number of compounds ^44^.

## Results

**Figure 2.**
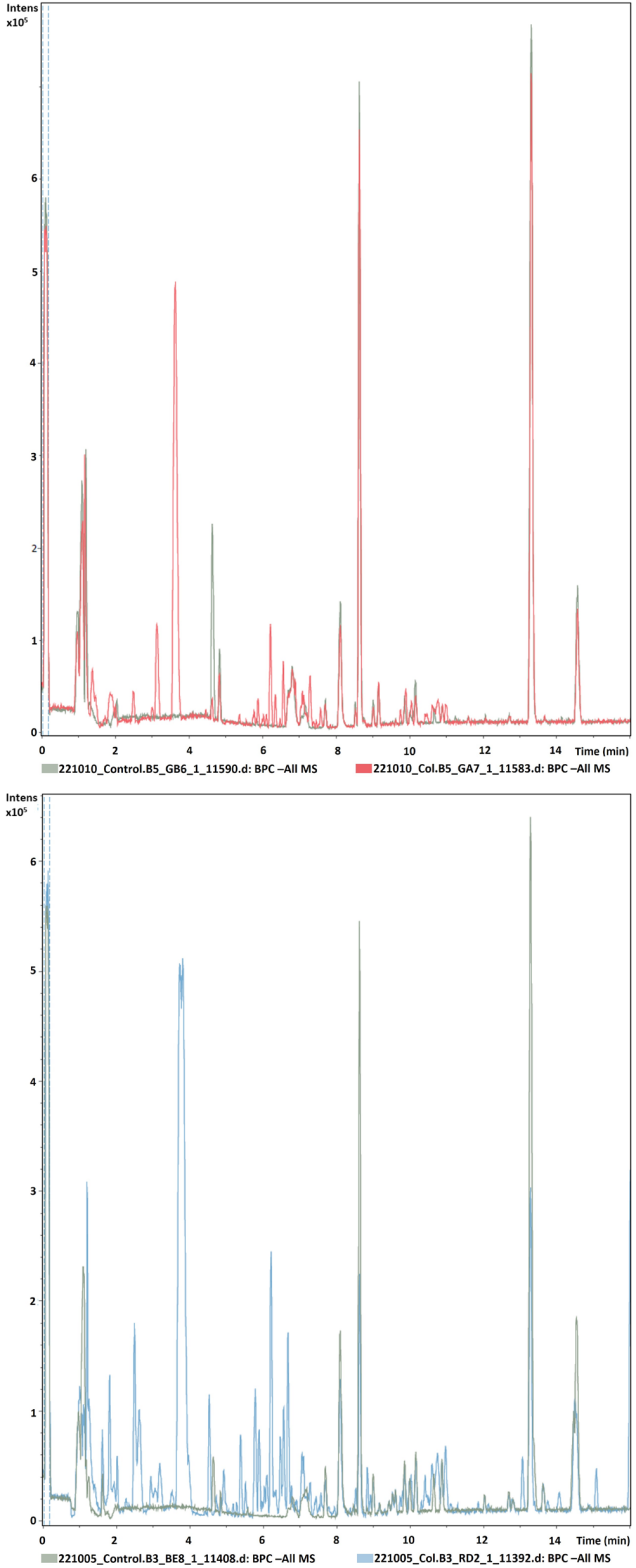
Representative chromatograms of two different Col-0 accessions (m/z 121–832, rt 0.99–15 minutes) obtained from root exudates overlaid over blank samples by UHPLC/ESI-QTOFMS in negative mode.

Out of the 35 Col-0 phytometers that were used in the experiment, 28 were successfully sampled for root exudates. The remaining plants either did not survive or were too small to be sampled. Figure 2 shows the chromatograms obtained from the untargeted analysis in negative ionization mode of Col-0 accessions using the above-described method overlaid over controls (in green). MZmine3 identified and aligned 378 features/chemical compounds in the 28 Col-0 samples, whereas the 10 random controls consisted of 68 features (see *Underlying data* ^45^). After subtracting the features present in controls, we identified 354 metabolic features present only in Col-0 accessions (see Supplementary Table 1 in *Extended data* ^45^). The CSI:FingerID web service feature of SIRIUS 5 classified 254 compounds (around 71% of the features identified) in the samples. Their chemical annotations are listed in Supplementary Table 2 (see *Extended data* ^45^).

Using the phenotypic data collected during the experiment (number of leaves and rosette size of plants at the time of sampling), we observed a clear correlation between plant size and total peak area ( *see Extended data analysis*) validating the ability of the methodology to accurately capture and quantify differences in compound abundance ^46, 47^. We also estimated the coefficient of variation (CV) and mean abundance for the detected compounds ( *see Supplementary Table 1*). We then calculated the relationship between CV and mean abundance based on the Poisson distribution, as suggested by Ipsen and Ebbels ^48^, and found it to be in line with the predicted relationship for LCMS data ( *see Extended data analysis*).

## Discussion

Root exudates are substantially hard to study, yet the importance of root-secreted signaling chemicals in mediating plant interactions is no longer debatable. So far, the methodologies available to trap and identify root-secreted signaling chemicals have remained a serious problem ^49^. This has limited our ability to identify any patterns of their natural variation on a large sample of accessions, and thus, their role in driving belowground communications remains to be elucidated.

The method developed here was tuned for large-scale root exudate sampling of *A. thaliana*, natural accessions. With this method, by reducing the time of collection and omitting orbital shaking, we aimed to diminish the shock effects experienced by plants during transfer from solid to liquid media. Furthermore, faster chemical analysis time is another significant improvement when handling hundreds of samples. Even with minute sampling solutions, the method yielded sufficient signals for the LCMS analysis, which was sensitive enough to capture microquantities of several metabolites, considering the sample sizes ( *i.e.,*smaller plants showed smaller total peak areas, while larger plants exhibited larger total peak areas). This is further supported by the number of secondary metabolites identified in the analysis. Assessment of reproducibility of the detected compounds through the coefficient of variation (CV) and mean abundance of detected compounds, further validates the robustness and reliability of our approach. Moreover, SIRIUS 5 was able to annotate a substantial number of features (listed in Supplementary Table 2 in *Extended data* ^45^), and this information can be used to draw broad patterns and make inferential hypotheses to be validated by complementary approaches. Our method is valuable for quick pattern assessment experiments of large samples of accessions for the purpose of studying plant-environment interactions (including but not limited to plant-microbes, plant-insects, and plant-plants).

Even though hydroponic root exudate collection is not a perfect solution, as we cannot fully mimic the conditions roots may experience in nature, and there can be potential variations in the exudate profiles derived from hydroponic *vs*. soil environments ^12^, these methods are still highly useful setups that allow capturing exudates in sterile environments ^13^. In this method, using a cost-effective plastic floater, plants were easily managed during the entire growth and hydroponic sampling steps. Furthermore, this floating setup could be translated into longer temporal collection experiments, as in similar experiments conducted on *A. thaliana* ^19, 24^. Using meshes of different pore sizes, one can customise this setup for root exudate collection and analysis of several individual genotypes from other plant species.

Verifying the biochemical functions of these metabolites is without a doubt, a mammoth feat, requiring an interdisciplinary approach. Analyzing root exudates in axenic conditions gives a broad overview of their chemical composition, and they can be further validated for their roles using complementary methodologies spanning multiple disciplines. For example, compounds tentatively identified by SIRIUS 5, can be quantified using a targeted approach if authentic standards are available. Many root exudate studies have previously confirmed an altered pattern of root behavior once their ability to sense the exudates of neighboring plants is removed ^3^. Targeting specific components of exudates for validating this behavior could also present an interesting next step. Deep investigation into the biochemical pathways of these metabolites by creating gene knockouts could also reveal their exact roles in plant behavior modulation. As a preliminary validation step, we successfully confirmed the accuracy of SIRIUS5 within our team by targeting the identification and quantification of standards, such as ketopinic acid, tryptophan and cyclic GMP, for a subset of samples analyzed in this study ^50^.

Studying root exudate chemical signals mediating belowground interactions are important not only from an ecological context but also from an applied perspective in agriculture and forestry ^1^. Root-secreted compounds are part of a complex interaction network incorporating other inter/intraspecific neighbors, bacteria, fungi and invertebrates etc., where each interacting plant might receive individual signals from multiple sources, making this exceedingly difficult to dissect. Hence, methodologies are needed to first simplify studying these interactions and identify ecologically relevant biochemical communication channels ^51^, which can be ultimately applied in agricultural context ^1^. Scrutinizing the variation in chemical profiles of interacting partners remains an essential first step, which has been largely overlooked in root exudate studies ^13^, further hindered by following a targeted approach to identify already known compounds.

The method developed here takes a step forward in overcoming limitations of previously developed hydroponic systems. By developing an easy-to-manage setup for largescale sampling, we detected many interesting secondary metabolites in Col-0, which will be used for further investigations using population genomics, evolutionary ecology and analytical chemistry approaches to answer questions relating to their roles in plant-environment interactions.

## Ethics and consent

Ethical approval and consent were not required.

## Data Availability

Zenodo: Unraveling the secrets of plant roots: Simplified method for large scale root exudate sampling and analysis in Arabidopsis thaliana. https://doi.org/10.5281/zenodo.8004460 ^45^. This project contains the following underlying data: Raw datafiles.xlsx: The raw datasets obtained from MZmine 3 analysis, which contains aligned features of Columbia genotypes (Sheet1) as well as control samples (Sheet2). The dataset consists of *feature (row)ID, average (mass-to-charge ratios) m/z* and *retention times (RT)* across samples for individual features. It also includes sample-specific information including *feature status, name, m/z, RT, feature peak height,* and *area*. We also include a filtered datasheet excluding the features obtained in control as well as samples (Sheet3). Sheet 4 contains the phenotypic data on the number of leaves and rosette size of the 28 replicates at the time of sampling, along with the total peak area for each sample from MZmine data. Zenodo: Unraveling the secrets of plant roots: Simplified method for large scale root exudate sampling and analysis in Arabidopsis thaliana. https://doi.org/10.5281/zenodo.8004460 ^45^. This project contains the following extended data: Supplementary tables-.xlsx: The 354 metabolites obtained after filtering out control features are listed with their *mass-to-charge ratios*, *retention times, and the mean, variance and coefficient of variation of the peak areas*(Supplementary Table 1). Supplementary Table 2 details the features identified by SIRIUS 5with their *mass-to-charge ratios, retention times, chemical formula, chemical annotations,* and corresponding *probabilities scored by SIRIUS* Extended data analysis: Contains supporting data analysis for reproducibility and validity of our method for root exudate collection and analysis in *Arabidopsis thaliana.* These extended data analyses enhance the understanding of the relationship between plant phenotypic traits, peak area, and variation in compound abundance Data are available under the terms of the Creative Commons Attribution 4.0 International license (CC-BY 4.0).
