# Supplementary material for: Unraveling the secrets of plant roots: Simplified method for large scale root exudate sampling and analysis in Arabidopsis thaliana
Source: Open Res Eur. 2023 Oct 20;3:12. Originally published 2023 Jan 18. [Version 3] doi: 10.12688/openreseurope.15377.3 (PMC10445920; doi:10.12688/openreseurope.15377.3)
Supplement: Peer review history

# Reviewer report: Reviewer response for version 3

**DOI:** 10.21956/openreseurope.18104.r35757

**Published:** 2023-10-26

**Authors:** Sophie Alvarez

**Affiliations:** 1 Department of Agronomy and Horticulture, University of Nebraska-Lincoln, Lincoln, Nebraska, USA

The authors have appropriately addressed the comments.

Is the rationale for developing the new method (or application) clearly explained?

Yes

Is the description of the method technically sound?

Partly

Are the conclusions about the method and its performance adequately supported by the findings presented in the article?

No

If any results are presented, are all the source data underlying the results available to ensure full reproducibility?

Yes

Are sufficient details provided to allow replication of the method development and its use by others?

Partly

Reviewer Expertise:

Plant stress, root exudate, metabolomics, proteomics, mass spectrometry

I confirm that I have read this submission and believe that I have an appropriate level of expertise to confirm that it is of an acceptable scientific standard.

# Reviewer report: Reviewer response for version 2

**DOI:** 10.21956/openreseurope.17459.r33537

**Published:** 2023-07-21

**Authors:** Sophie Alvarez

**Affiliations:** 1 Department of Agronomy and Horticulture, University of Nebraska-Lincoln, Lincoln, Nebraska, USA

Thanks to the authors for addressing the comments. However I still have some questions. 

1. I see the authors have included time information for the steps, but can they also include what the overall time is from beginning of sample collection to transferring samples to vials?
2. Can the authors please use the word 'annotation' or 'annotated' instead of 'identification' or 'identified' for the metabolite. Based on the supplementary information from the "identification", the annotation has clearly not been reviewed or confirmed with authentic standards, so these are only putative identifications. There are redundant annotations, and annotations not endogenous to plants. 
3. Is it also unclear what the confidence scoring reported in the table is, and if there was any threshold used for any of the probabilities reported by NPC, Classyfire or SIRIUS?
4. Finally, how do authors explain the presence of a large number of compounds belonging to fatty acids in their samples, if the collection type used here is meant to be for polar compounds. 

Is the rationale for developing the new method (or application) clearly explained?

Yes

Is the description of the method technically sound?

Partly

Are the conclusions about the method and its performance adequately supported by the findings presented in the article?

No

If any results are presented, are all the source data underlying the results available to ensure full reproducibility?

Yes

Are sufficient details provided to allow replication of the method development and its use by others?

Partly

Reviewer Expertise:

Plant stress, root exudate, metabolomics, proteomics, mass spectrometry

I confirm that I have read this submission and believe that I have an appropriate level of expertise to confirm that it is of an acceptable scientific standard, however I have significant reservations, as outlined above.

# Response

**Published:** 2023-10-17

**Authors:** Harihar Jaishree Subrahmaniam

**Affiliations:** Ecoscience, Aarhus University, Aarhus, Denmark

We are grateful for the additional questions from Reviewer 1 which enable us to clarify important aspects of our methodology further. Below we have pasted our “point-by-point” response to the comments.  

**Reviewer 1 follow-up questions** Thanks to the authors for addressing the comments. However I still have some questions.

1. I see the authors have included time information for the steps, but can they also include what the overall time is from beginning of sample collection to transferring samples to vials?
2. Can the authors please use the word 'annotation' or 'annotated' instead of 'identification' or 'identified' for the metabolite. Based on the supplementary information from the "identification", the annotation has clearly not been reviewed or confirmed with authentic standards, so these are only putative identifications. There are redundant annotations, and annotations not endogenous to plants.
3. Is it also unclear what the confidence scoring reported in the table is, and if there was any threshold used for any of the probabilities reported by NPC, Classyfire or SIRIUS?
4. Finally, how do authors explain the presence of a large number of compounds belonging to fatty acids in their samples, if the collection type used here is meant to be for polar compounds.

**Response to Reviewer 1 comments**

**Overall time for sample collection to vial transfer:** Thank you for emphasizing the importance of a comprehensive timeframe. We have updated the Methods section to provide a timeframe from beginning of sample collection to transferring samples to vials, taking into account the 120 minutes allocated for exudate collection. This brings the total estimated time to a range of 133-136 minutes per sample.

**Use of 'annotation' instead of 'identification':** We appreciate your insightful suggestion to use 'annotation' in lieu of 'identification' for greater accuracy. Given that the metabolites have not been confirmed by comparison with authentic standards, the term 'annotation' more appropriately reflects the level of confidence in these putative identifications. Accordingly, the manuscript has been revised to make these adjustments throughout.

**Confidence scoring in tables:** We understand that the confidence scoring presented in our tables might have been ambiguous. These scores are raw values sourced directly from the NPC, ClassyFire, and SIRIUS databases, and no additional thresholding has been applied. We believe presenting the unfiltered data underscores our commitment to methodological transparency. In the Results section, we have also incorporated a statement about the 'Confidence Level 3' classification for our annotated metabolites, as per the framework by Blazenovic et al. (2018).

**Presence of fatty acids in samples:** The observation regarding the prevalence of fatty acids in our samples is indeed pertinent. We employed a Waters Acquity UHPLC HSS T3 column, which is a C18 reverse-phase column designed for high selectivity and peak shape across a wide polarity range. While the column is capable of separating compounds with a broad range of polarities, it is particularly effective for non-polar to moderately polar compounds, including fatty acids. Unlike a hydrophilic interaction liquid chromatography (HILIC) column, which is more focused on the separation of polar compounds, our setup does not selectively exclude less polar molecules like fatty acids, particularly when considering our chosen mobile phase conditions.

 We trust that these modifications adequately address the concerns and questions raised by Reviewer 1 and serve to augment the rigor and clarity of our study.

 Sincerely, Harihar Jaishree Subrahmaniam on behalf of all authors

# Reviewer report: Reviewer response for version 2

**DOI:** 10.21956/openreseurope.17459.r33535

**Published:** 2023-07-11

**Authors:** Mònica Escolà Casas

**Affiliations:** 1 Department of Environmental Chemistry, IDAEA-CSIC, Barcelona, Spain

I have no further comments to make.

Is the rationale for developing the new method (or application) clearly explained?

Partly

Is the description of the method technically sound?

Partly

Are the conclusions about the method and its performance adequately supported by the findings presented in the article?

Partly

If any results are presented, are all the source data underlying the results available to ensure full reproducibility?

Yes

Are sufficient details provided to allow replication of the method development and its use by others?

Partly

Reviewer Expertise:

I am an environmental scientist working with nature based solutions (constructed wetlands, biofilms) for the removal of contaminants of emerging concern in water. Lately I have been investigating how root exudates are affected by contaminants of emerging concern and how root exudates may help to their degradation.

I confirm that I have read this submission and believe that I have an appropriate level of expertise to confirm that it is of an acceptable scientific standard.

# Reviewer report: Reviewer response for version 1

**DOI:** 10.21956/openreseurope.16622.r31315

**Published:** 2023-06-06

**Authors:** Paul A Weston

**Affiliations:** 1 Charles Sturt University, Wagga Wagga, New South Wales, Australia

This manuscript presents an interesting approach to quantifying and identifying metabolites from *Arabidopsis thaliana* and potentially many other plant species. The proposed method offers many advantages over other techniques that have been used previously, but there are some shortcomings in their presentation of the methods and interpretation of results. In addition, there are numerous grammatical errors that require correction.

 The major shortcomings are as follows:

1. The authors provide no indication of the plant-to-plant variation in metabolite profiles. This is not a trivial concern because most metabolomic studies pool samples collected from numerous individuals to smooth out plant-to-plant variation in order to focus on larger trends in metabolite variation. Is the greater investment in time and resources involved with growing and sampling individual plants justified by the results? We can’t tell because the authors provide no information on this variation.
2. Collecting exudates by suspending seedlings in pure water might significantly affect the exudates produced by the seedlings. How relevant are these exudates to those that might be produced by the seedlings in soil? How does the duration of the collection impact the quality and quantity of exudate produced? Incidentally, one must assume the collection liquid is water, although the authors refer to the medium in the collection vessels somewhat mysteriously as “MilliQ sampling solution” on page 6.
3. The anaerobic conditions arising from exclusion of air from the collection vessels and preventing aeration of the sampling medium might be expected to significantly alter the quantity or quality (or both) of exudates produced by the seedlings. The magnitude of this effect would be influenced by the collection interval; was the collection interval examined to determine if this effect was significant?
4. It is unclear how a glove can be used to determine the level of vacuum in the desiccator.

Regarding the more mundane issue of correctness of expression, the authors use the terms aboveground and belowground inconsistently (sometimes each is presented as two words, sometimes one, and sometimes with a hyphen). Regardless of which style the authors decide on, they should use the wording consistently throughout the manuscript. As noted on the manuscript, a space should be used between numerals and units of measurement. Capitalization of numerous words (e.g. Sodium, Liquid chromatographs, etc.) is done incorrectly. I also thought the wording "to absorb any erroneous exudation pattern" on page 4 was very poor.  I think you mean to say something such as "extraneous exudates", that is exudates produced by the plants prior to the sampling interval. The word "erroneous" is inappropriate to use as well; these exudates might be extraneous, but can't be said to be erroneous.

 More substantively, the chromatograms in Figure 2 would be easier to see if they were compressed vertically and if the legend text was larger. Related to this figure, it is difficult to distinguish between the control and treatment chromatogram in the left panel; readers with red/green color blindness will have no hope of distinguishing the two traces, which is challenging even for readers with normal color vision.

Is the rationale for developing the new method (or application) clearly explained?

Yes

Is the description of the method technically sound?

Partly

Are the conclusions about the method and its performance adequately supported by the findings presented in the article?

Partly

If any results are presented, are all the source data underlying the results available to ensure full reproducibility?

Yes

Are sufficient details provided to allow replication of the method development and its use by others?

Partly

Reviewer Expertise:

Metabolomics, natural products chemistry, entomology, plant physiology

I confirm that I have read this submission and believe that I have an appropriate level of expertise to confirm that it is of an acceptable scientific standard, however I have significant reservations, as outlined above.

# Response

**Published:** 2023-06-27

**Authors:** Harihar Jaishree Subrahmaniam

**Affiliations:** Ecoscience, Aarhus University, Aarhus, Denmark

We would like to thank the reviewer for their valuable feedback on our manuscript. We have thoroughly reviewed all the comments and suggestions and incorporated substantial revisions to address them. The revised manuscript includes track changes to highlight all the modifications made. Reviewer 3 raised several important points that have helped us address the potential limitations of our methodology and inconsistencies in the manuscript. We have addressed these points individually.  

**Comment 1:** The authors provide no indication of the plant-to-plant variation in metabolite profiles. This is not a trivial concern because most metabolomic studies pool samples collected from numerous individuals to smooth out plant-to-plant variation in order to focus on larger trends in metabolite variation. Is the greater investment in time and resources involved with growing and sampling individual plants justified by the results? We can’t tell because the authors provide no information on this variation.

**Response: **We acknowledge the importance of considering plant-to-plant variation in metabolite profiles. To address this concern, we performed data analysis using established methods, including calculating the coefficient of variation (CV) and mean abundance for the detected compounds in the 28 replicates. The results of the data analysis, including CV and mean abundance, are now included in the *Extended data analysis* section of the revised manuscript. These additions provide insights into the reproducibility and plant-to-plant variability of the detected compounds across samples.  

**Comment 2:** Collecting exudates by suspending seedlings in pure water might significantly affect the exudates produced by the seedlings. How relevant are these exudates to those that might be produced by the seedlings in soil? How does the duration of the collection impact the quality and quantity of exudate produced? Incidentally, one must assume the collection liquid is water, although the authors refer to the medium in the collection vessels somewhat mysteriously as “MilliQ sampling solution” on page 6.

**Response: **We agree that collection of exudates by suspending seedlings in pure water may influence the exudates produced by the seedlings compared to those produced in soil. We address the potential limitations and relevance of exudates collected using this method in the *Introduction* and the *Discussion* section. Additionally, on page 6, we removed the term "MilliQ sampling solution" and clarify that the term refers to sampling vials containing MilliQ water.  

**Comment 3:** The anaerobic conditions arising from exclusion of air from the collection vessels and preventing aeration of the sampling medium might be expected to significantly alter the quantity or quality (or both) of exudates produced by the seedlings. The magnitude of this effect would be influenced by the collection interval; was the collection interval examined to determine if this effect was significant?  

**Response:** We acknowledge the possible impact of anaerobic conditions and collection interval on the quantity and quality of exudates. Although our study did not examine the influence of collection interval specifically, our protocol was consistent with our previous time exposure experiments on another plant species and similar studies that suggest brief sampling periods to maintain sterility and reduce stress times. We now provide an account of these studies in the *Introduction* our revised manuscript.  

**Comment 4:** It is unclear how a glove can be used to determine the level of vacuum in the desiccator.

**Response:** In the revised manuscript, we address how a glove can be used to determine the level of vacuum in the desiccator. We briefly explain in the *Methods *section how a glove indicator is used as a visual cue to assess the level of vacuum inside a sealed container like a desiccator. As the vacuum pump removes air from the desiccator, the pressure inside decreases, causing the glove attached to the desiccator to expand. The expansion of the glove indicates that the desired level of vacuum has been achieved.  

**Comment 5:** Regarding the more mundane issue of correctness of expression, the authors use the terms aboveground and belowground inconsistently (sometimes each is presented as two words, sometimes one, and sometimes with a hyphen). Regardless of which style the authors decide on, they should use the wording consistently throughout the manuscript. As noted on the manuscript, a space should be used between numerals and units of measurement. Capitalization of numerous words (e.g. Sodium, Liquid chromatographs, etc.) is done incorrectly. I also thought the wording "to absorb any erroneous exudation pattern" on page 4 was very poor. I think you mean to say something such as "extraneous exudates", that is exudates produced by the plants prior to the sampling interval. The word "erroneous" is inappropriate to use as well; these exudates might be extraneous, but can't be said to be erroneous.

**Response: **We have addressed inconsistencies in terminology, capitalization, and grammatical errors to ensure adherence to scientific language conventions. The term "erroneous" has been replaced with "extraneous" for more accurate representation.  

**Comment 6:** More substantively, the chromatograms in Figure 2 would be easier to see if they were compressed vertically and if the legend text was larger. Related to this figure, it is difficult to distinguish between the control and treatment chromatogram in the left panel; readers with red/green color blindness will have no hope of distinguishing the two traces, which is challenging even for readers with normal color vision.

**Response:** We have refined Figure 2 by compressing the chromatograms vertically and increasing the size of the legend text for enhanced visibility. Additionally, we have adjusted the color schemes for better differentiation between the control (green) and sample chromatograms (in blue and red).

# Reviewer report: Reviewer response for version 1

**DOI:** 10.21956/openreseurope.16622.r32231

**Published:** 2023-05-30

**Authors:** Mònica Escolà Casas

**Affiliations:** 1 Department of Environmental Chemistry, IDAEA-CSIC, Barcelona, Spain

This method article describes how to sample and analyze root exudates from Arabidopsis Thaliana by using a plastic float and a small volume of water. The analysis is made by UPLC-qTOF using a common methodology. Finally, the data curation and treatment are thoroughly done using different softwares. With this approach the authors can capture a large number of polar and semi-polar root-metabolites with minimal disturbance of the plant when transferring it to the sampling trap.

 The article is well written, but it lacks some improvement in the following points:

 1) The introduction should be more clear in the sense that it should follow a logical order of ideas. Also, some concepts are repeated over the paragraphs (e.g. the belowground signaling process and its importance in plants is mentioned several times). Finally, a few examples of significant root-sampling methods should be presented and compared with the method that they are about to present in their study.

 2) As it is a method paper, a description of the method should put emphasis on why their approach is better than the approaches of other methods. E.g. "previous studies have found difficulties sampling in xxx way so we decided to investigate xxx possibility and found that xxx was the best solution".

 3) The description of the method is well done but some specific details are missing:

 3.1 What was the composition of the plastic mesh (type of plastic)? Define the type of plastic mesh and maybe the supplier should be included as it may be replicated.

 3.2 What was the shape of the 15mL glass vials (400 uL in a 15 mL vial sounds like a very small height of the liquid and very limited space for the roots). Maybe Figure 1 could include measures of the vials.

 3.3 The article mentions biological replicates. How were they made? Only plastic mesh with no plant?

 3.4 The sampling method is crucial in this article. In this sense, the authors have chosen a 2h-collection in Milli-Q water. Have the authors tried other exposure times? Also, sampling in ultrapure water might have affected exudation by stress. The authors should dedicate some sentences explaining that (although it is a common practice).

 4) Why did the authors chose the ESI negative ionization mode?

 5) In the results, the authors mention that there were a few hundred chemical annotations. Have the articles verified any of the annotations by injecting some true standards? I know the goal here was not to identify compounds but it could add some value to the article if you could mention that some of the annotations were verified with a true standard.

Is the rationale for developing the new method (or application) clearly explained?

Partly

Is the description of the method technically sound?

Partly

Are the conclusions about the method and its performance adequately supported by the findings presented in the article?

Partly

If any results are presented, are all the source data underlying the results available to ensure full reproducibility?

Yes

Are sufficient details provided to allow replication of the method development and its use by others?

Partly

Reviewer Expertise:

I am an environmental scientist working with nature based solutions (constructed wetlands, biofilms) for the removal of contaminants of emerging concern in water. Lately I have been investigating how root exudates are affected by contaminants of emerging concern and how root exudates may help to their degradation.

I confirm that I have read this submission and believe that I have an appropriate level of expertise to confirm that it is of an acceptable scientific standard, however I have significant reservations, as outlined above.

# Response

**Published:** 2023-06-27

**Authors:** Harihar Jaishree Subrahmaniam

**Affiliations:** Ecoscience, Aarhus University, Aarhus, Denmark

We would like to thank the reviewer for their valuable feedback on our manuscript. We have thoroughly reviewed all the comments and suggestions and incorporated substantial revisions to address them. The revised manuscript includes track changes to highlight all the modifications made. Reviewer 2 raised several important points that have helped us improve the logical order and several details of our methodology. We have addressed these points individually.  

**Comment 1:** The introduction should be more clear in the sense that it should follow a logical order of ideas. Also, some concepts are repeated over the paragraphs (e.g. the belowground signaling process and its importance in plants is mentioned several times). Finally, a few examples of significant root-sampling methods should be presented and compared with the method that they are about to present in their study.

**Response: **We have restructured the *Introduction* section to follow a logical order of ideas and eliminate the repetition of concepts. We have also highlighted examples of significant root-sampling methods and compared them with the method presented in our study. These revisions aim to provide a clearer and more organized introduction that effectively introduces the novelty and importance of our approach.  

**Comment 2:** As it is a method paper, a description of the method should put emphasis on why their approach is better than the approaches of other methods. E.g. "previous studies have found difficulties sampling in xxx way so we decided to investigate xxx possibility and found that xxx was the best solution".

**Response: **In response to the reviewer's comment, we have emphasized why our approach is superior to other methods. We now provide an explanation in each subsection of the *Introduction *section, highlighting the challenges faced by previous methods that led us to develop more effective solutions.  

**Comment 3:** The description of the method is well done but some specific details are missing:

1.
  1. What was the composition of the plastic mesh (type of plastic)? Define the type of plastic mesh and maybe the supplier should be included as it may be replicated

**Response: **The plastic mesh used in our study was made of polypropylene. We have now included this information in the *Introduction *section of the revised manuscript to ensure clarity and replicability.

1.
  1. What was the shape of the 15mL glass vials (400 uL in a 15 mL vial sounds like a very small height of the liquid and very limited space for the roots). Maybe Figure 1 could include measures of the vials.

**Response: **In the revised version of the manuscript, we have provided a description of the shape and dimensions of the vials in the *Methods *section of the revised manuscript. The glass vials used in the study had a capacity of 20 mL and a cylindrical shape with a height of 57 mm and a diameter of 27.5 mm. Despite the limitations in terms of root space for larger plants, the short collection time span allowed us to still collect sufficient exudates.  

1.
  1. The article mentions biological replicates. How were they made? Only plastic mesh with no plant?

**Response: **The biological controls consisted of plastic meshes without plants. We have revised the manuscript to explicitly mention this and provide a clearer explanation of the controls used and their relevance for comparative analysis and validation of our methodology in the beginning of the *Methods* section.

1.
  1. The sampling method is crucial in this article. In this sense, the authors have chosen a 2h-collection in Milli-Q water. Have the authors tried other exposure times? Also, sampling in ultrapure water might have affected exudation by stress. The authors should dedicate some sentences explaining that (although it is a common practice).

**Response: **We have addressed the importance of the sampling method and the choice of a 2-hour collection in ultrapure water in the *Introduction* of the revised manuscript. We explain that this duration is a common practice and briefly discuss the potential effects of stress caused by exposure to ultrapure water during the collection process. We also mention our previous time exposure experiments on another plant species and other references to support this collection time.  

**Comment 4:** Why did the authors chose the ESI negative ionization mode?

**Response: **Although we conducted analysis in both positive and negative ionization modes, we presented the results in the negative mode as it captured a greater number of compounds. This information is included in the manuscript at the end of the *Methods *section to enhance the understanding of our analytical approach.  

**Comment 5:** In the results, the authors mention that there were a few hundred chemical annotations. Have the articles verified any of the annotations by injecting some true standards? I know the goal here was not to identify compounds but it could add some value to the article if you could mention that some of the annotations were verified with a true standard.

**Response: **While our primary focus was not the annotation of compounds, we have recently undertaken a targeted approach to validate the chemical compounds we identified in the exudates. We now mention this in the *Discussion* section of the revised manuscript, highlighting the efforts made to verify the annotations using true standards.

# Reviewer report: Reviewer response for version 1

**DOI:** 10.21956/openreseurope.16622.r31027

**Published:** 2023-04-12

**Authors:** Sophie Alvarez

**Affiliations:** 1 Department of Agronomy and Horticulture, University of Nebraska-Lincoln, Lincoln, Nebraska, USA

The authors present a new methodology for root exudate collection and analysis for *Arabidopsis *using a hybrid agar-hydroponic system to grow the plants and collect the root exudate. The aim of the new methodology described is to reduce handling of the roots to minimize damage that could bias the composition of the root exudate. The method also reduced the number of steps to collect concentrated root exudate from one individual plant for further analysis using mass spectrometry.

 Although, the methodology sounds very simplified for a better representation of the composition of the root exudate. There are still some questions that authors have left unanswered to validate the relevance and reproducibility of the method.

1. The authors mentioned using paintbrush to clean the roots from agar before the washing/equilibrium step included before collection. It seems that damage and stress could still occur at that stage. Also, authors should indicate the time it takes to go through the steps from transfer of the plants from plates to the collection and storage of root exudates, to evaluate/estimate the feasibility of larger experiments. Can all the 28 replicates be done within the same time period of the day, so diurnal changes are not a variable in the experiment?
2. There is no justification for the 2 hours sample collection. Was a time course done to evaluate that 2 hours was the minimum required to get the levels high enough to be detected by untargeted LC-MS, but not detrimental to the plant (cell leakage or damage)?
3. The authors share the data from the detected compounds with peak area, but did not perform any data analysis of the reproducibility of the data collected from the 28 replicates to evaluate the experimental variation of the methodology, and to also determine how many technical replicates would be necessary to compare a large population of Arabidopsis.  There is no correlation included between the data collected from the root exudate and the possible phenotype observed (in the case plants have “chlorosis”). This is essential to be able to validate their methodology.
4. There is no description of what the biological controls used to compare the data from Col0 are. What’s the relevance of it? Please explain in the manuscript.
5. It is also difficult to evaluate the methodology and its relevance with regards to identification and quantification of the root exudate content using an untargeted LC-MS metabolomics approach, when the authors voluntarily excluded the annotation of the compounds in the extended data.

Minor comments:

1. The method section is poorly written compared to the rest of the manuscript.
2. There is a typo in Figure 1. “recorded”

Is the rationale for developing the new method (or application) clearly explained?

Yes

Is the description of the method technically sound?

Partly

Are the conclusions about the method and its performance adequately supported by the findings presented in the article?

No

If any results are presented, are all the source data underlying the results available to ensure full reproducibility?

Yes

Are sufficient details provided to allow replication of the method development and its use by others?

Partly

Reviewer Expertise:

Plant stress, root exudate, metabolomics, proteomics, mass spectrometry

I confirm that I have read this submission and believe that I have an appropriate level of expertise to state that I do not consider it to be of an acceptable scientific standard, for reasons outlined above.

# Response

**Published:** 2023-06-27

**Authors:** Harihar Jaishree Subrahmaniam

**Affiliations:** Ecoscience, Aarhus University, Aarhus, Denmark

We would like to thank the reviewer for their valuable feedback on our manuscript. We have thoroughly reviewed all the comments and suggestions and incorporated substantial revisions to address them. The revised manuscript includes track changes to highlight all the modifications made. Reviewer 1 raised several important points that have helped us improve the clarity and reproducibility of our methodology. We have addressed these points individually:    

**Comment 1:** The authors mentioned using paintbrush to clean the roots from agar before the washing/equilibrium step included before collection. It seems that damage and stress could still occur at that stage. Also, authors should indicate the time it takes to go through the steps from transfer of the plants from plates to the collection and storage of root exudates, to evaluate/estimate the feasibility of larger experiments. Can all the 28 replicates be done within the same time period of the day, so diurnal changes are not a variable in the experiment?

**Response: **We acknowledge the concern regarding potential damage and stress during the cleaning step using a paintbrush. To minimize any bias introduced at this stage, we allowed the roots to remain in the cleaning solution for 1-2 minutes, as suggested by Oburger and Jones (2018) to capture any extraneous root exudation caused by stress. Additionally, we have provided detailed information in the *Methods* section on the time required to go through each step of the methodology, including the transfer of plants from plates to the collection and storage of root exudates. We confirm that all 28 replicates can be performed within the same time period of the day to minimize diurnal changes as a variable in the experiment.  

**Comment 2: **There is no justification for the 2 hours sample collection. Was a time course done to evaluate that 2 hours was the minimum required to get the levels high enough to be detected by untargeted LC-MS, but not detrimental to the plant (cell leakage or damage)?

**Response:** In the revised version, we have included references in the *Introduction* to support the use of the 2-hour sampling period based on previous studies and briefly mention our previous time exposure studies on a different plant species to support the collection period. These references provide evidence that 2 hours is sufficient to capture root exudates at detectable levels without causing exudate re-uptake or detrimental effects such as cell leakage or damage to the plants.  

**Comment 3:** The authors share the data from the detected compounds with peak area, but did not perform any data analysis of the reproducibility of the data collected from the 28 replicates to evaluate the experimental variation of the methodology, and to also determine how many technical replicates would be necessary to compare a large population of Arabidopsis.  There is no correlation included between the data collected from the root exudate and the possible phenotype observed (in the case plants have “chlorosis”). This is essential to be able to validate their methodology.

**Response: **In the revised manuscript, we have now included data analysis of the reproducibility *(in Extended data analysis)*, including the coefficient of variation (CV) and mean abundance for the detected compounds. We also calculated the relationship between CV and mean abundance based on the Poisson distribution, as suggested by Ipsen and Ebbels (2012), and found it to be in line with the predicted relationship for LCMS data. Moreover, we have discussed the correlation between plant size and total peak area as empirical evidence validating the methodology's ability to capture and quantify differences in compound abundance in the *Discussion* *section*.  

**Comment 4:** There is no description of what the biological controls used to compare the data from Col0 are. What’s the relevance of it? Please explain in the manuscript.

**Response: **We acknowledge the lack of description regarding the controls used to compare the data from Col0. In our study, we used plastic meshes without plants as controls. These control meshes were treated in the same manner as the plants during sample preparation and analysis. They served as a baseline reference for comparison, allowing us to assess the specific contribution of root exudates from Col0. We have included an explanation of controls in the beginning of the *Methods *section. We acknowledge that the term "biological controls" may have caused confusion, and we have revised the manuscript to provide a clearer explanation of the controls used and their relevance for comparative analysis and validation of our methodology. We apologize for any confusion caused and appreciate the opportunity to clarify this in the revised manuscript.  

**Comment 5:** It is also difficult to evaluate the methodology and its relevance with regards to identification and quantification of the root exudate content using an untargeted LC-MS metabolomics approach, when the authors voluntarily excluded the annotation of the compounds in the extended data.

**Response: ** We have now provided the full chemical annotations of the identified compounds in the *Supplementary data file, *which includes a comprehensive list of the identified compounds along with their annotations  

**Minor comments:**

1. The method section is poorly written compared to the rest of the manuscript.
2. There is a typo in Figure 1. “recorded”

**Response:** We have also addressed the minor comments, particularly the method section, and made substantial revisions to improve its clarity and coherence. Furthermore, we have corrected the typographical error in Figure 1 as pointed out by the reviewer, ensuring that the revised version of the figure accurately represents the recorded data.  
